# The splenium of the corpus callosum: embryology, anatomy, function and imaging with pathophysiological hypothesis

**DOI:** 10.1007/s00234-019-02357-z

**Published:** 2020-02-15

**Authors:** J. Blaauw, L. C. Meiners

**Affiliations:** 1 Department of Radiology, University Medical Center Groningen, University of Groningen, 9700 RB Groningen, The Netherlands; 2 Faculty of Medical Sciences/Department of Neurology, University Medical Center Groningen, University of Groningen, 9700 RB Groningen, The Netherlands

**Keywords:** Splenium, Corpus callosum, MRI, Anatomy, Pathophysiology, Consciousness

## Abstract

**Background and purpose:**

The splenium of the corpus callosum is the most posterior part of the corpus callosum. Its embryological development, anatomy, vascularization, function, imaging of pathology, possible pathophysiological mechanisms by which pathology may develop and the clinical consequences are discussed.

**Methods:**

A literature-based description is provided on development, anatomy and function. MR and CT images are used to demonstrate pathology. The majority of pathology, known to affect the splenium, and the clinical effects are described in three subsections: (A) limited to the splenium, with elaboration on pathophysiology of reversible splenial lesions, (B) pathology in the cerebral white matter extending into or deriving from the splenium, with special emphasis on tumors, and (C) splenial involvement in generalized conditions affecting the entire brain, with a hypothesis for pathophysiological mechanisms for the different diseases.

**Results:**

The development of the splenium is preceded by the formation of the hippocampal commissure. It is bordered by the falx and the tentorium and is perfused by the anterior and posterior circulation. It contains different caliber axonal fibers and the most compact area of callosal glial cells. These findings may explain the affinity of specific forms of pathology for this region. The fibers interconnect the temporal and occipital regions of both hemispheres reciprocally and are important in language, visuospatial information transfer and behavior. Acquired pathology may lead to changes in consciousness.

**Conclusion:**

The development, location, fiber composition and vascularization of the splenium make it vulnerable to specific pathological processes. It appears to play an important role in consciousness.

## Introduction

The splenium is the most posterior and bulbous shaped part of the corpus callosum (CC). Anterior to the splenium, the remainder of the CC consists, respectively, of the narrow isthmus, the thicker corpus, the voluminous genu, with the thinnest part, called the rostrum, extending inferiorly to the anterior commissure.

The CC forms the bridge between the cerebral hemispheres, containing crossing axonal fibers from both hemispheres.

The fibers in the splenium are projections from the occipital-parietal and temporal cortex [1].

During the embryological phase, the development of the hippocampi and hippocampal commissure (HC) precedes the development of the CC. After developmental completion, the splenium has an intimate connection anteriorly with the HC [1].

The exact function of the splenium is not completely understood, but a splenial lesion may result in the disconnection of the cerebral hemispheres, with disruption of higher cortical function, loss of conscious processes and delirious behavior [2].

This article focuses on the embryological development of the CC, and the splenium in particular, together with that of the closely related HC. Furthermore, its normal anatomy, function and its vascularization are discussed.

The last section provides an overview of the majority of congenital and acquired splenial pathology, as seen on MRI and CT. We have categorized this section into three subsections to facilitate reading. The first subsection discusses pathology restricted to the splenium, with elaboration on pathophysiology of reversible splenial lesion syndrome (RESLES). The second subsection focuses on CNS diseases affecting the posterior parietal-occipital-temporal white matter, including metabolic disease, tumors and inflammatory disease, which may extend into or derive from the splenium, with special emphasis on tumors. The third subsection discusses generalized disorders, such as trauma, infarction and intracranial hypotension syndrome, which may specifically involve the splenium.

## Embryological development of the CC and hippocampus and their postnatal anatomy

The development of the CC has long been believed to take place in a fixed order, starting with the genu during the 12th gestational week, followed by the isthmus, the splenium, and finally the rostrum during the 18–20th week of gestation [3, 4]. In 2010, Raybaud, however, hypothesized that the formation of the CC is based on fusion of separate segments. Anteriorly, containing the axons from the anterior hemisphere, and those from the posterior neocortex forming the splenium. This fusion hypothesis makes certain subtypes of CC agenesis, associated with other developmental brain disorders easier to understand [1].

There is a clear developmental relation of the CC with the hippocampus and hippocampal commissure. At week 7, a primary joining plate between the hemispheres, referred to as the lamina reuniens, starts to thicken. In the anterior part, fibers of the anterior commissure (AC) start to cross. In the dorsal part, the massa commisuralis is formed and becomes the bed for the ingrowth of commissural fibers of the CC [1, 4]. From 10 weeks, hippocampal-septal fibers develop, forming the early fornix. At 11 weeks, some of the fornix fibers cross the midline in the dorsal part of the lamina reuniens and form the HC [1, 4]. At 11–12 weeks, pioneer fibers of the prospective CC begin to penetrate the massa commisuralis in the primordium hippocampi, and at 12–13 weeks, an increasing number of fibers form the definitive commissural plate [4]. At 13 weeks, cingulate fibers and other neocortical fibers start forming the anterior CC and other neocortical fibers cross via the AC and HC. At 13–14 weeks, the anterior CC and splenium start to fuse, forming the entire CC [1].

Growth of the CC is associated with reduction of the hippocampal formation in the frontal lobe [5]. Kier et al. found that at 13 weeks, a large part of the medial surface of the cerebral hemisphere is occupied by the hippocampal formation, which runs along a wide hippocampal sulcus or fissure, extending from the olfactory tract, located in the frontobasal area, to the temporal lobe, forming the inner limbic arch [5].

At 14 weeks, during the development of the CC, the supracallosal hippocampus starts to regress, with the induseum griseum, also known as the supracallosal gyrus, overlying the CC, remaining as a remnant. The sulcus of the CC is a remnant of the embryological hippocampal sulcus and is located between the cingulate gyrus and the supracallosal gyrus. At 16 weeks, the cingulate gyrus and the temporal parahippocampal gyrus can be identified. Together with the subcallosal area, they form the outer limbic arch.

**Fig. 1:**
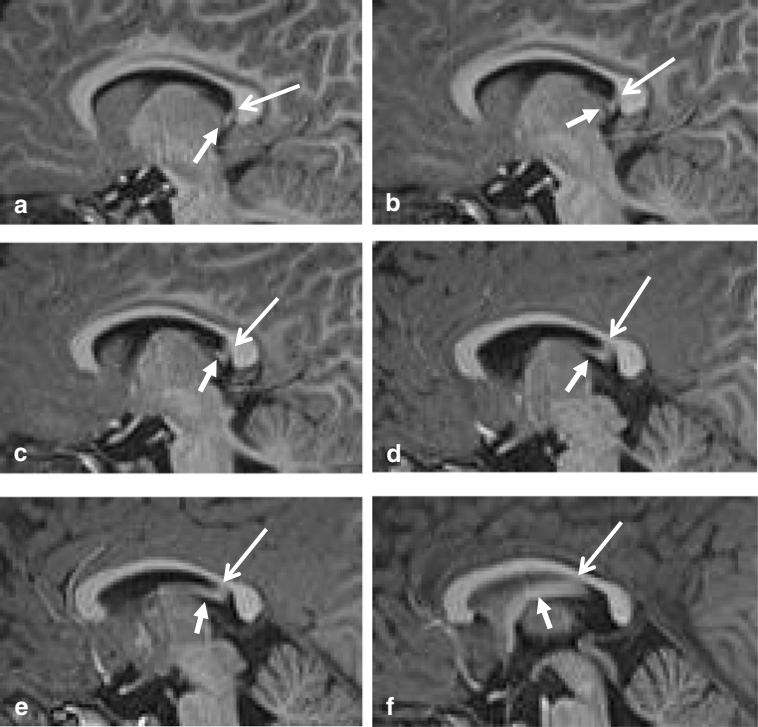
Sagittal 3D T1-weighted image (**a**–**f**) with short closed arrow pointing at the fornix and long open arrow indicating the hippocampal commissure, extending from the ventral splenium to the isthmus

The CC grows more rapidly than the HC, and eventually, the splenium overrides the HC [4], which has become a triangular structure between the fornices, with a midline attachment to the undersurface of the splenium [1], as demonstrated in Fig. 1. In humans, the HC is usually very thin; however, in some cases, an enlarged HC may be mistaken for the splenium on a sagittal view [6].

The fibers of the HC and the splenial fibers, connecting the posterior parietal, the inferior temporal and occipital cortices of the two hemispheres, cross the midline together [1, 7]. A schematic drawing of the HC is provided by Ranson and Clark [5, 8]. Because of their intimate relationship and development, splenial agenesis may be associated with an agenesis or possibly an anterior shift in location of the HC, explaining the bilateral malrotation of hippocampi often seen in these cases [9].

After birth, the growth rate of the splenium exceeds that of the genu. At 8 months of age, the midsagittal splenium area achieves 55% of the average adult size [10].

Most splenial fibers are thought to be reciprocal and connect the hemispheres homotopically. De Lacoste et al. showed that callosal connections from the temporo-parietal-occipital junctional region course through the splenium and the caudal part of the body of the CC [11]. Neuroanatomical tracer studies have shown that together with the genu, the anterior and mid splenium contain the largest density of thin (> 0.4 μm) axonal fibers, connecting higher order processing areas of the parietal and medial temporal lobes. Thicker fibers (> 3–5 μm) are present in the posterior splenium, fusing the hemirepresentation of the visual field [12].

Between the age of 7 and 11 years, the splenium remains comparatively stable. In the age group of 11–15 years, relatively more rapid splenial growth occurs compared with the anterior CC [13]. The increase may be related to increased oligodendroglial proliferation associated with the larger diameter fibers, rather than increased axonal density [12]. A DTI study in adolescents and young adults has shown that in the splenium, until 18 years, age correlates with increased white matter integrity and therefore myelination, leading to increased fractional anisotropy [14]. During puberty, splenial size increase could facilitate maturing of multiple higher functions, such as reading and calculation skill, requiring visuospatial information transfer, allowing these functions to expand [15–17].

## Arterial vascularization and venous drainage

The arterial vascularization of the splenium, based on examination of 30 adult human brains, is well illustrated by Kahilogullari et al. [18]. The splenium receives its blood supply from three arteries: the anterior pericallosal artery, which is a terminal branch of the anterior cerebral artery. The posterior pericallosal artery (branch of the posterior cerebral artery), also known as the splenial artery. Lastly, from the posterior accessory pericallosal artery. These arteries ramify into perforating branches, and the branches of both arteries anastomose to form a pericallosal pial plexus [18, 19]. Inside the CC, these arteries distribute numerous terminal or collateral branches, which run between the nervous fibers. They anastomose with homologous neighboring branches to form a vascular network, which is closely connected with the commissural fibers [20].

A detailed description of the venous drainage of the CC is provided by Wolfram-Gabel and Maillot [21]. They illustrate two drainage systems in a schematic drawing. The main system consists of callosal veins and venules and of callosocingulate veins. The splenium usually contains short callosal veins, which run downwards perpendicular to the central surface of the CC. The callosocingulate veins emerge from the peripheral surface to form long callosal veins, which drain the CC and cingulate gyrus. These veins join together at the central surface of the CC, to form the subependymal veins. Those in the posterior third of the CC drain into the septal veins and medial atrial vein, and eventually into the internal cerebral veins. Wolfram-Gabel and Maillot also describe an accessory drainage system, comprising the posterior pericallosal veins and the splenial veins, draining into the anterior straight sinus, into the vein of Galen, the basal vein, medial atrial vein or into the medial occipital vein.

## Function

### Transferring eloquent information between both hemispheres

Up to 1940, the neurological function of the CC was not understood. Callosotomy, used in the treatment of epilepsy, first reported in 1940, has allowed a better understanding of its function. It has led to the view that the CC is involved in transferring information between the cerebral hemispheres [15]. The splenium has been shown to be involved in visuospatial information transfer, language, reading and calculation scores, IQ, behavior and consciousness [15–17]. Fractionated anisotropy, the measure of axonal directionality, has been shown to have a positive correlation with processing speed, expressive vocabulary and single-word reading. Several studies have found splenial enlargement in dyslexia and reduction in attention-deficit hyperactivity disorder [22].

### Disconnection syndrome

A known major side effect of a callosotomy, transecting the entire CC, is the so-called split brain or disconnection syndrome. The disconnection syndrome is a combination of disorders in cortical function such as alien limb syndrome, apraxia, tactile and/or visual anomia, agraphia neglect and dyslexia, and it is often of a transient nature [23]. Section of the splenium leads to a sensory disconnection syndrome, which expresses itself with neglect of visual stimuli presented only to the right or left visual field, if the verbal access to this information is interrupted. This has been proposed to result from isolating the dominant language hemisphere from visual information received by the non-dominant hemisphere [24], which can be explained by the primary visual and temporo-occipital and parietal association commissural fibers found in the splenium. Using fMRI and DTI, Fabri et al. found foci evoked by auditory and visual stimuli in the isthmus/splenium continuum and in the splenium itself. They suggest that proximal body representation is provided by callosal fibers running through the posterior isthmus and anterior splenium, transferring combined occipital located visual notion and parietal localized spatial representation [25]. These microanatomical and functional findings may explain why splenial lesions may be associated with confusion and most commonly altered mental status, with more specific findings of splenial compromise being hallucinations, psychosis and mutism [26].

### Consciousness

With splenial lesions, the HC may also be affected, considering its close relation with the splenium. The exact function of the HC is not clarified. However, it might serve as a memory sluice, permitting information initially processed in the temporal lobe cortex of one hemisphere to be further processed partially in the contralateral hippocampus or to share specific memories which are predominantly dependent on one temporal lobe [27]. Damage to the hippocampal commissure, combined with the loss of visuospatial and auditory information transfer in splenial pathology and further disintegration of the cerebral network, may lead to changes in consciousness.

Using DTI, Zhang et al. determined that consciousness levels correlated strongly with a reduction of fractional anisotropy value in the corpus of the CC and moderately in the splenium, corresponding with increased demyelination [28]. Acute severing of the crossing fibers following head trauma has been shown to lead to altered mental status and even coma [29]. Furthermore, posttraumatic lesions in the splenium, combined with those in the dorsal brainstem, have been shown to be highly significant in predicting non-recovery in patients with a posttraumatic vegetative state [30].

## Pathology affecting the splenium

Several articles have provided a pictorial overview of pathology affecting the entire CC [31–33]. However, few have been published on pathology solely affecting the splenium. In this article, pathology in the splenium is separated in three subsections: (A) acquired pathology primarily affecting the splenium, (B) acquired parietal-occipital-temporal cerebral pathology extending into or from the splenium and (C) congenital and acquired pathology involving the splenium in a general disorder.

### A. Acquired pathology primarily affecting the splenium

#### A1.Reversible splenial lesions

Transient, reversible lesions in the splenium have been described in a wide range of disorders, including viral, bacterial and parasitic infections, treatment of infection with metronidazole, anti-epileptic drug (AED) toxicity or withdrawal, treatment with 5 fluorouracyl, hypoglycemia, hyponatremia and high altitude cerebral edema [31, 32, 34–36]. Similar lesions have been described on MRI in patients with Wernicke syndrome, with persisting cognitive impairment following thiamine replacement. It is, however, not known whether these splenial lesions were reversible, as no follow-up imaging was published [37, 38]. Furthermore, a case has been published with intravenous immunoglobulin therapy related reversible diffusion restriction in the entire splenium, extending into the parietal white matter [39].

The clinical-radiological condition associated with these lesions has been termed ‘reversible splenial lesion syndrome’ (RESLES) [34], ‘mild encephalitis/encephalopathy with a reversible splenial lesion’ (MERS) [40], ‘boomerang lesion’ [41] and recently cytotoxic lesions of the CC that show restricted diffusion (CLOCCs) [42]. We prefer the term RESLES, as the clinical presentation associated with the lesion is not always mild, as implied by the term MERS. CLOCCs does not necessarily imply reversibility and boomerang sign does not indicate the splenial location. In this section, we will use the term RESLES and MERS depending on the articles to which are referred.

#### *MR imaging in RESLES*

**Fig. 2:**
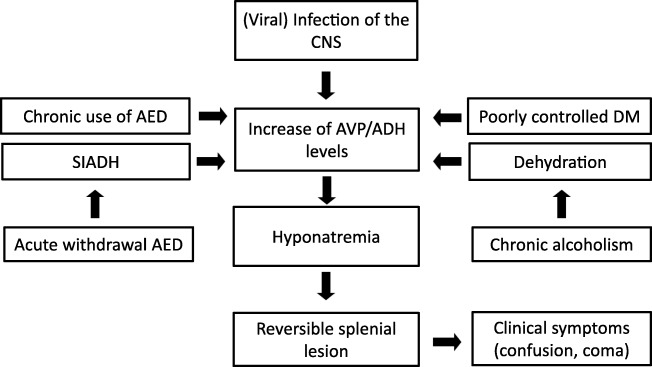
The flowchart provides a possible mechanism by which a reversible splenial lesion may develop from differents pathological processes leading to hyponatremia

On MRI, during the acute phase of RESLES, localized signal intensity increase is seen on DWI with a reduced signal on the ADC map, consistent with cytotoxic edema [42–44]. The abnormal area is slightly hyperintense on T2 and FLAIR, and hypointense on T1, surrounded by normal crossing axons [31]. Contrast enhancement has only been published in one patient, treated prophylactically with AEDs. The authors suggested the enhancement to be due to more intense focal damage, confirmed by the presence of a residual lesion on follow-up [45]. This is also shown in Fig. 2d.

In adults, the reversible lesion is always located in the centre of the splenium, with an unsharp border and never extending laterally, and this has been referred to as MERS type 1 lesion [46, 47].

In children, lesions may also be small as demonstrated in Fig. 2a, which shows a lesion on DWI located in the center of the splenium in a girl with an entorovirus infection. Figure 2 b–d show MR images of a girl who was admitted with pneumoccocal meningitis with a larger splenial lesion on initial T2 and a small rightsided residual lesion on 5-year follow-up. Several articles have published more extensive lesions, extending into the entire CC and into the parietal white matter, and sometimes even into the frontoparietal white matter [42, 46, 48], also referred to as MERS type 2 lesions [46].

Timing of development and disappearance of RESLES lesions varies. In infections, lesions have been described to be visible from day 1 after presentation of clinical symptoms and disappearing within 1–2 weeks in most [34]. Following AED withdrawal, lesions have been found coincidentally between 24 h and 1 week. In patients who continued to use a AEDs, RESLES lesions have been shown up to 3 weeks after a last seizure [34].

#### *Pathophysiological considerations*

Several pathophysiological theories have been proposed for the development of transient splenial lesions.

One hypothesis focuses on the possibility that it may be related to arginine-vasopressin (AVP)/antidiuretic hormone (ADH) level. In 1956, Nyhan and Cooke already suggested that in CNS infections, hyponatremia may develop because of acute expansion of the extracellular fluid volume, which may follow increased production of ADH, limiting the capacity to excrete water in these patients [49]. In response to a brain injury such as infection or trauma, increased levels and enhanced effect of ADH may occur, referred to as syndrome of inappropriate secretion of ADH (SIADH) [50, 51].

In the kidney, ADH binds to receptors, which trigger an intracellular mechanism that opens AQP2 water channels allowing water to be reabsorbed from the urine into the cell. Opening of the AQP3 and AQP4 water channels at the basolateral cell membrane leads to reabsorption of intracellular water into the blood. This leads to hypervolemia, resulting in dilution and decrease in plasma osmolality, therefore hyponatremia. For a more detailed description, we refer to Verbalis et al. [52].

Sodium ions are the major cations of the extracellular fluid and potassium ions are the major cations of the intracellular fluid. To maintain internal fluid and electrolyte balance, water, sodium and potassium are in constant movement between both compartments, regulated by the sodium-potassium pump. The most important function of this pump is preventing cells from swelling. If sodium is not ‘pumped’ out, water accumulates within the cell, which causes swelling and ultimately bursting [53]. In the brain, this movement of water from the extracellular space into the cells in response to this osmotic gradient, results in cerebral edema.

It is a known fact that rapid correction of acute severe hyponatremia may lead to pontine myolinolysis [54]. With hyponatremia, the cells mostly involved in swelling are glial cells. Specific aquaporin water channels (AQP1 and AQP4) allow water to pass into the glial cells, whereas neurons are relatively spared from water entry [2, 51, 55]. Besides cytotoxic edema occuring at glial level, which has also been suggested by Prilipko et al [56], intramyelinic cytotoxic edema has also been proposed as a cause for a RESLES lesion [44, 57, 58].

The preference for a RESLES lesion to develop in the center of the splenium is unknown. Because of the bigger volume of the splenium compared to the remainder of the CC, the center contains the most compact area of callosal glia cells combined with a known largest density of thin (> 0.4 μm) axonal fibers in the anterior and mid splenium [12]. In case of insidious development of uncorrected hyponatremia, the larger density of AQP1 and AQP4 channels and a higher density of glutamate receptors [42] may make this area most vulnerable to water influx in glial cells and development of cytotoxic edema.

In 2009, Takanashi et al. showed that 25/30 patients with MERS had hyponatremia [59]. In 2015, the same group showed that five patients with MERS after mumps vaccination and mild encephalitis, all had hypotonic hyponatremia [55].

Although these findings underscore a relation between hyponatremia and RESLES lesions, a very limited number of cases have been described with hypernatremia [42]. Tsuji et al. described a case with influenza A infection scanned on day six, which had neither hypo- nor hypernatremia, presenting with an asymptomatic RESLES lesion with diffusion restriction [60].

Opposed to diffusion restriction seen in the acute stage of ischemia, which nearly always leads to irreversible damage if untreated, the reversibility of diffusion restriction and of behavioral symptoms, in a RESLES lesion has been explained by a transient inflammatory response following sustained hypo-osmolality. Inflammatory cytokines trigger a cascade, whereby massively increased amounts of glutamate enter the extracellular space and glutamate reuptake is blocked. The highly increased extracellular glutamate level leads to excitotoxic effect on certain receptors triggering the sodium-potassium pump, allowing sodium ions to enter cells and potassium ions to leave cells. This results in entering of water in glial cells and in neurons, resulting in cytotoxic edema and diffusion restriction [42, 51].

#### *Pathology and RESLES*

**Fig. 3:**
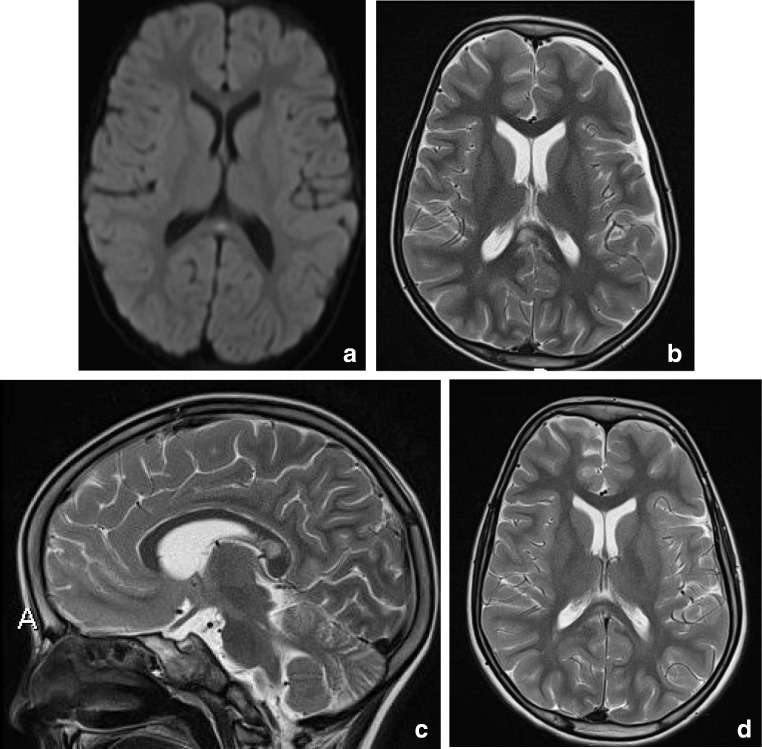
(a) Three-year-old girl with gastroenteritis by enterovirus. Axial DWI (**a**) on day 4 shows a small lesion with unsharp border consistent with a RESLES lesion. (b-d) Six-year-old girl with a pneumococcal meningitis following an ear infection. Axial and sagittal T2 at admission (**b**, **c**) shows an area of edema in the splenial and dorsal isthmus (no DWI was made). At 5-year follow-up, a small residual lesion is seen on the axial T2 (**d**)

RESLES lesions have been described in infections, the use and withdrawal of antiepileptic drugs, in alcoholism and in hypoglycemia, all of which have been related to ADH and AVP. (Figure 2) In a literature review, Garcia-Monco et al. found 38 patients with RESLES with an encephalopathy in the setting of a systemic infection, without CSF parameters of infection and without seizures or AED treatment. Influenza A and B virus and human herpes virus are the most commonly found in the presence of RESLES [46, 61]. It has also been reported in a patient with Dengue fever [61], and in patients with mumps, adenovirus, rotavirus and streptococcal and *E. coli* bacteria [40]. In Fig. 3a, an MRI of a female patient with an entorovirus infection is presented. She was admitted on day 3 with several convulsions and fever. On day 4, she developed diminished consciousness, which she regained on day 7. In Fig. 3b–d, the MRI scan of a girl with pneumococcal meningitis is shown. She had limited contact with the outside world during the time of scanning. Clinically, 4 months after the meningitis, she had no cognitive deficits. Five years later, however, she developed behavioral changes, and on MRI, a small rightsided residual splenial lesion was seen.

**Fig. 4:**
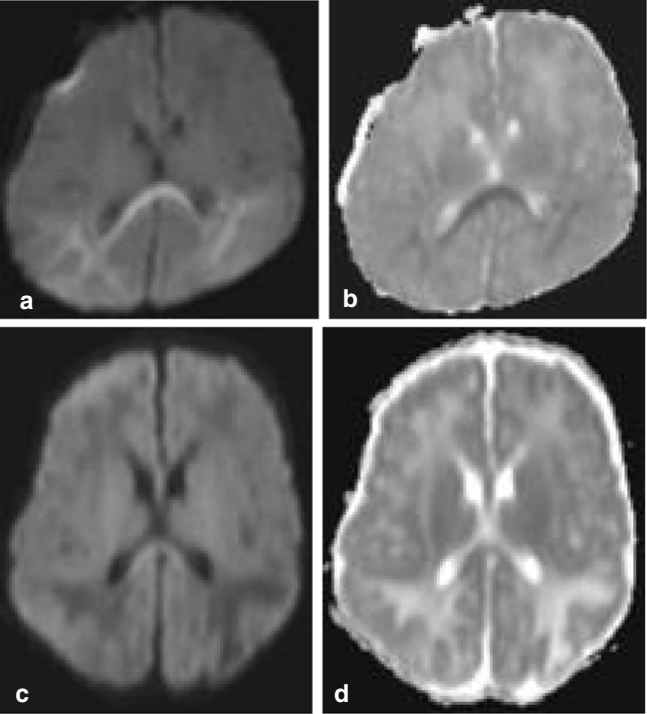
Seven-day-old child born at 38 + 5 weeks with a difficult delivery and an APGAR score of 4–5–6 was admitted to another hospital. It was transferred to our hospital because of seizures at 2 days postpartum. On admission, the child had a hypoglycemia (< 0.5 mmol/l) for which glucose infusion was given. DWI (**a**) and ADC (**b**) made 5 days after admission showed diffusion restriction in the splenium and in the subcortical white matter of the parietal-occipital lobes. On follow-up DWI (**c**) and ADC (**d**) made 16 days after admission, the signal intensity of the splenium normalized, with remaining white matter edema in the parietal lobes and widening of the ventricles, due to global tissueloss

In epileptic patients, AVP disbalance or enhancement of the antidiuretic effect, caused by chronic use of certain anti-epileptic drugs, such as phenytoin, carbamazepine and lamotrigine, may lead to hyponatremia and cerebral edema [34, 60, 62]. On the other hand, abrupt withdrawal of AEDs may cause a short period of disequilibrium, causing a syndrome of inappropriate antidiuresis. This could contribute to brain edema with splenial diffusion restriction representing cytotoxic edema [43, 51, 62], and perilesional increased signal on T2 and FLAIR, consistent with vasogenic edema [43, 53]. RESLES lesions do not seem to be caused by seizures, independent of type and frequency [34]. This is underscored by the finding that these lesions have also been described in three non-epileptic patients treated with AEDs [45].

In alcoholism, callosal abnormalities, particularly in the splenium and the genu, have been attributed to vitamin B deficiency [63]. However, in our opinion, severe alcohol consumption causes chronic dehydration and loss of electrolytes, such as sodium, which together with increases in ADH, results in hyponatremia.

Hypoglycemia may also be associated with RESLES lesions in neonates [64], and in adults [65]. AVP also appears to play a role in glucose homeostasis, insulin resistance and diabetes mellitus (DM). It is markedly elevated in patients with poorly controlled DM and therefore may result in hyponatremia [66]. Figure 4 illustrates an MRI of a 5-day-old neonate with reversible splenial diffusion restriction caused by hypoglycemia.

**Fig. 5:**
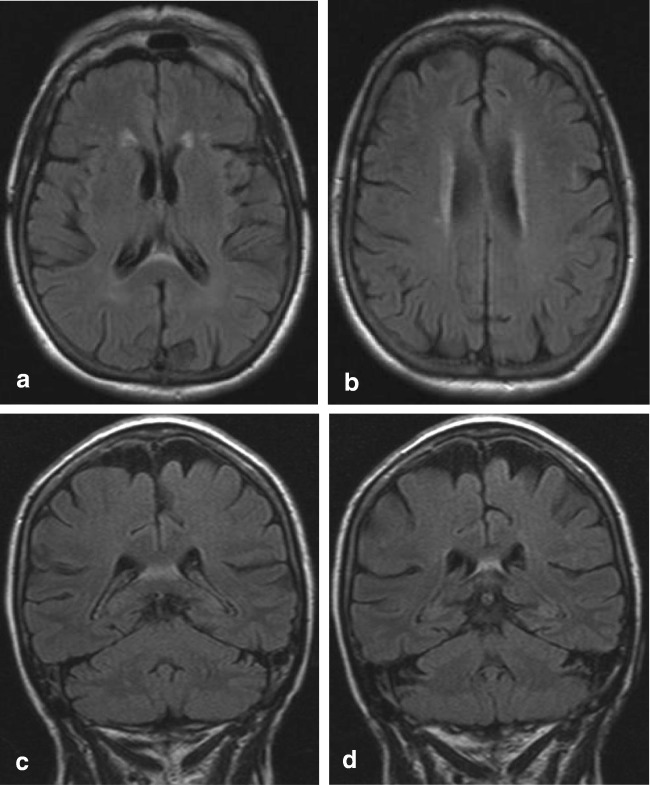
Seventy-six-year-old woman. Axial FLAIR (**a**, **b**) and coronal FLAIR (**c**, **d**). Hyperintense band ventral to the splenium in the region of the hippocampal commissure extending into the fornices

#### *Clinical presentation of RESLES*

The most common prodromal symptom of RESLES is fever. Patients with RESLES lesions may present without clinical symptoms, as was found in a large retrospective study by Kim et al. involving 1200 epileptic patients scanned with MRI. An asymptomatic lesion was found in six subjects, scanned 3–6 weeks following their last seizure [67]. RESLES lesions after AED withdrawal are also not always symptomatic [34].

Delirium is, however, the most commonly observed clinical symptom in over half of patients, followed by disturbed consciousness and seizures, recovering within a month [2, 40]. Seizure activity may be explained by loss of small organic osmolytes such as glutamate, which may result in transient neurological abnormalities [51]. Other clinical manifestations are confusion, disorientation, ataxia, disconnection syndrome, dysarthria, headache, coma and hallucinations [40, 68], most of which are also described in the presence of hyponatremia [50].

Of patients with influenza, > 10% has been shown to have delirious behavior. In their study of 370 patients with influenza, Takanashi et al. found MERS lesions in 5 of 11 patients (3%) experiencing intermittent episodes of delirious behavior [2, 40].

Katoh et al. found that 4/70 patients with hypoglycemia showing RESLES on DWI, clinically presented with a disturbed consciousness [65].

The correlation between a RESLES lesion and symptom relief is controversial [69]. Although most RESLES lesions disappear following clinical remission and have a favorable outcome, two cases have been described in which these lesions persisted 6–9 months, independent of clinical improvement [69]. A delay in treatment may, however, may lead to irreversible and prolonged consciousness disturbance [65].

To summarize, we propose that RESLES lesions could be considered to represent an osmotic imbalance syndrome. It may result from correction of, or in the presence of a more insidious chronic hyponatremia, due to imbalance of AVP. Combined with excitotoxic effect of Glutamate, water is enabled to enter into neuronal cells, as suggested by Starkey et al. [42]. Why some patients remain asymptomatic, while others present with severe neurological symptoms, and in some patients, RESLES lesions remain present after clinical recovery is unknown. Possibly, this may be related to the speed at which the hyponatremia developed and the timing of the scan.

#### A2. Ventral splenial edema

In adults, a rim of hyperintense signal on FLAIR at the ventral border of the splenium has been described in older patients and those that have been treated with radiotherapy [70]. This finding is illustrated in Fig. 5, showing FLAIR images of an older patient with mood and behavioral changes and cognitive decline.

**Fig. 6:**
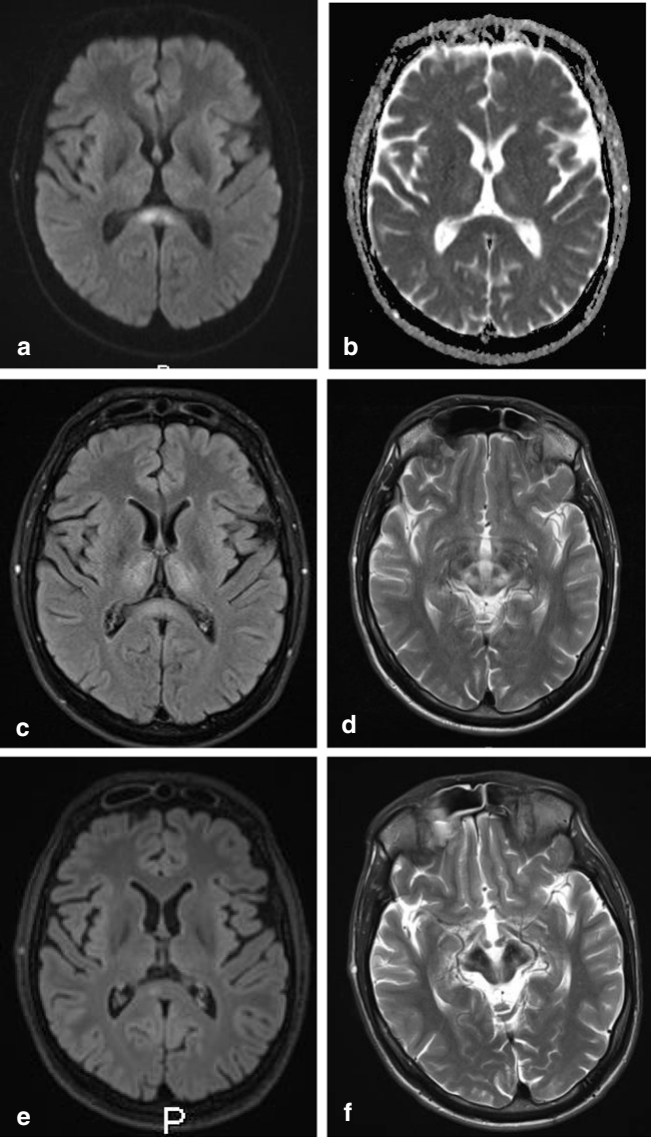
Thirty-year-old man. Axial DWI (**a**) showing a central area of high signal in the splenium, without low signal on ADC (**b**) and comparable high signal on FLAIR (**c**), compatible with T2 shine through effect and white matter edema. Axial T2 (**d**) shows a typical giant panda sign at the level of the mesencephalon-pons. On follow-up 2 years later, the DWI (**e**) shows a small residual splenial lesion on the right, and axial T2 (**f**) at the level of the mesencephalon shows atrophy and increase in low signal in the substantia nigra and nucleus ruber

Pekala et al. describe one autopsy case, which showed loss of axons and myelin sheaths and isomorphic gliosis. In their study, they found a clear correlation between this finding and white matter disease seen in normal aging [70].

We hypothesize that this region may represent edema in the hippocampal commissure. The pathological significance and the clinical relevance have not been studied so far and the precise effect on memory and behavior is unknown.

### B. Acquired parietal-occipital-temporal cerebral pathology extending into or from the splenium

#### B1.Metabolic diseases

Metabolic diseases, such as Metachromatic Leukodystrophy and Mucopolysaccharisodoses, usually affect the entire CC [32]. In Leighs disease, sole involvement of the splenium and no extension into the remainder of the CC is exceptional and has been described in only two case reports [71, 72]. In the following section, we will focus on (a) Wilson’s disease, in which the splenium may be affected; (b) X-linked adrenoleukodystrophy (X-ALD), which initially affects the splenium; and (c) Krabbe’s disease, which involves the splenium at a later stage, together with the peritrigonal white matter.

#### *B1a. Wilson’s disease*

Wilson’s disease (WD), also known as hepatolenticular degeneration, is an autosomal recessive disease of impaired copper metabolism, caused by a mutation in the ATP7B gene, encoding copper-transporting ATPase in the liver [73].

On MRI, brain lesions are usually bilateral and symmetrical and involve the basal ganglia, thalami, mesencephalon, pons and dentate nucleus, as shown in Fig. 5. Abnormal T2 signal in the splenium is not unusual and found in nearly a quarter of the patients [73, 74]. Trocello et al. describe that on follow-up, the splenial lesion does not change, noting that this is an argument against transient change [74]. However, the MRI follow-up in our patient showed only a residual splenial abnormality on DWI as shown in Fig. 6.

**Fig. 7:**
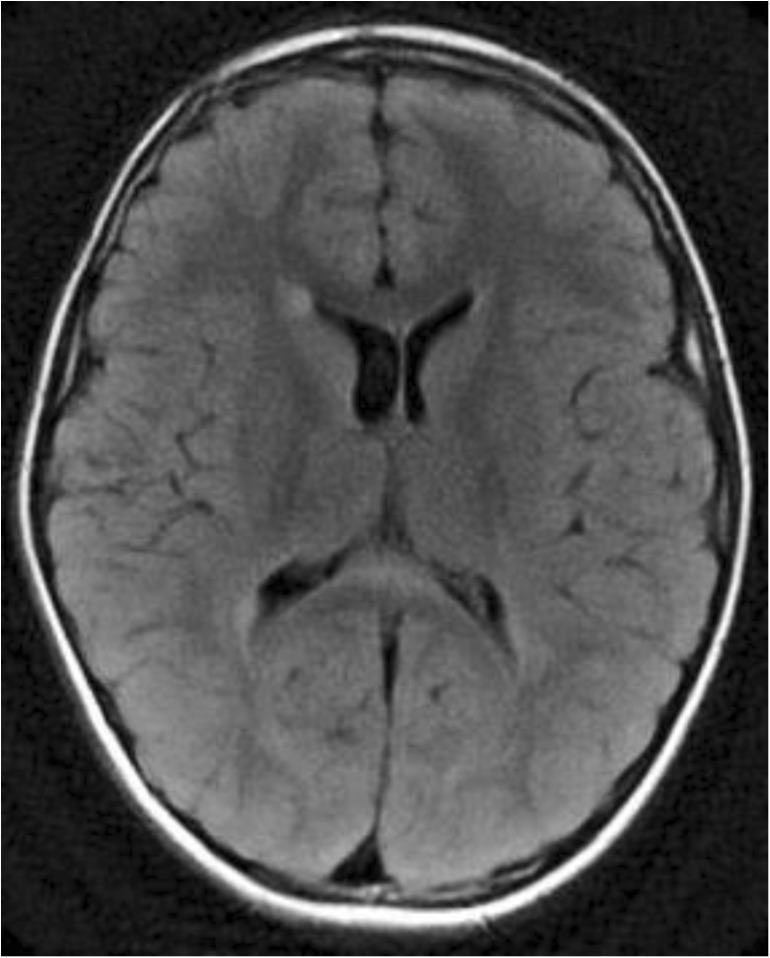
Seven-year-old boy with X-linked adrenoleukodystrophy. Axial FLAIR shows hyperintense area located in the ventral splenium

Clinically, these patients may present with neurological, psychiatric, liver and renal symptoms and corneal Kayser-Fleischer rings [73]. Although Trocello et al. describe that no clinical signs of disconnection were observed, possibly due to the slowly progressive nature, Zhou et al. report that WD patients with splenial lesions have more severe neurological and psychiatric dysfunction [73, 74].Our patient, a 30-year-old man presented with longstanding progressive cerebellar ataxia, dysarthria, swallowing disturbance, gait difficulties, bradyphrenia, memory deficits and autonomic disturbance.

#### *B1b. X-linked adrenoleukodystrophy*

X-linked adrenoleukodystrophy (X-ALD) is a peroxisomal disorder caused by a mutation in the ABCD1 gene [75]. In this disorder, fatty acids build up in the body. The accumulated fatty acids are particularly harmful and can destroy specific cells and organs, including the myelin sheathes of the brain.

In 80% of homozygote X-ALD patients, the initial demyelinating lesion may precede symptoms. On MRI, it is localized in the splenium and progresses into the adjacent parieto-occipital white matter [75–77]. The abnormalities are hyperintense on T2-weighted images and FLAIR, as shown in Fig. 7. The abnormalities may show variable contrast enhancement [32].

**Fig. 8:**
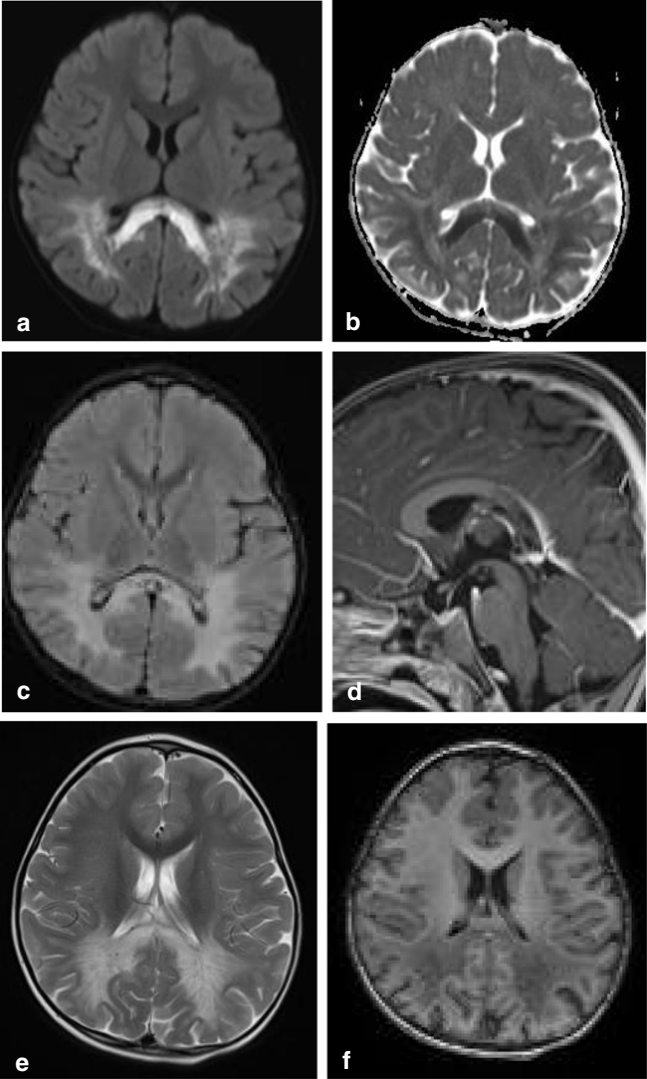
Three-year-old boy with Krabbe’s disease. Axial DWI (**a**) and ADC (**b**) show extensive abnormal signal with partial diffusion restriction involving the splenium and parietal white matter. Axial FLAIR (**c**) and sagittal T1 after gadolinium (**d**) show widened (hypointense) perivascular spaces in the splenium. Axial T2 (**e**) and inversion recovery (**f**) show spared axons in the parietal white matter

Clinically, children with X-ALD, initially present with visual difficulties, behavioral problems, hyperactivity and emotional lability. Our patient presented with behavioral disturbances, loss of cognitive functions and since several months development of abnormal gait. In general these symptoms are rapidly progressive, especially in the juvenile form. It may lead to death within 2–4 years or to a vegetative state, which could be explained by splenial involvement of the patient [76].

#### *B1c. Krabbe’s disease*

Krabbe’s disease, or globoid cell leukodystrophy, is an autosomal recessive lysosomal storage disease affecting the central nervous system, with a primary defect in the galactosyl-cerebrosidase enzyme. This defect leads to impaired degradation of acetosylceramide, resulting in apoptosis of myelin forming cells [78].

Brain pathology is characterized by globoid cells, which have multiple nuclei derived from microglia [79].

In adult Krabbe disease, on MRI, the corticospinal tracts and white matter in the parietal lobes are affected, with frequent involvement of the splenium. These findings are also present in a boy of which the MRI scan is shown in Fig. 8. The splenial preference may possibly be related to the presence of microglia fountains (see section B on lymphoma) [80].

**Fig. 9:**
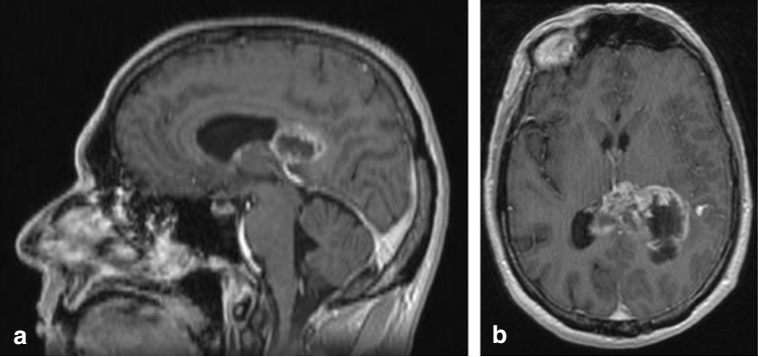
Fifty-year-old male with a glioblastoma multiforme. Sagittal contrast-enhanced T1 (**a**) and axial T2 (**b**) show irregular tumor mass with central necrosis and irregular enhancing rim in the splenium, extending into the left parietal white matter

Clinically, Krabbe’s disease often presents during early childhood with irritability, psychomotor regression and at the end stage the child becomes decerebrate with loss of contact with surroundings [81], which may be related to splenial involvement. Our patient with proven Krabbe’s disease, based on two mutations in the GALC gene, presented with progressive gait disturbance and visual disorder month before MRI, followed by panic attacks 2 weeks after MRI. One and a half years later he developed loss of contact with his surroundings.

#### B2. Tumors

Glioblastoma multiforme (GBM) and lymphoma are known to develop in or extend into the splenium or the remainder of the CC [82]. We would also like to add the diffuse infiltrating glioma (previously known as gliomatosis cerebri) to this list. As oligodendrogliomas often develop in the frontal lobes, they usually involve the genu of the CC if they cross the midline and therefore we have not included this tumor in the discussion [33].

The reason why only aggressive tumors involve the CC is not known. It has been suggested that the compact nature of the white matter tracts in the CC may make this structure relatively resistant to infiltration by edema or tumor spread [83].

#### *B2a. Glioblastoma multiforme and diffuse infiltrating glioma type tumor*

Glioblastoma multiforme (GBM) is the most aggressive diffuse astrocytic tumor (WHO grade IV). Its most common imaging appearance is that of a large heterogeneous mass in the supratentorial white matter, which may involve the splenium through direct extension along the white matter tracts [84, 85].

Diffuse glioma type tumor, formerly known as gliomatosis cerebri [86, 87], is a rare, diffusely growing malignant neuroepithelial tumor characterized by extensive brain infiltration, involving more than two cerebral lobes [88]. On T2-weighted images, diffuse gliomatous tumor infiltration in the splenium is less hyperintense than peritumoral vasogenic edema. Although splenial involvement is not specifically described in articles on gliomatosis, cases have been demonstrated in multiple article figures.

**Fig. 10:**
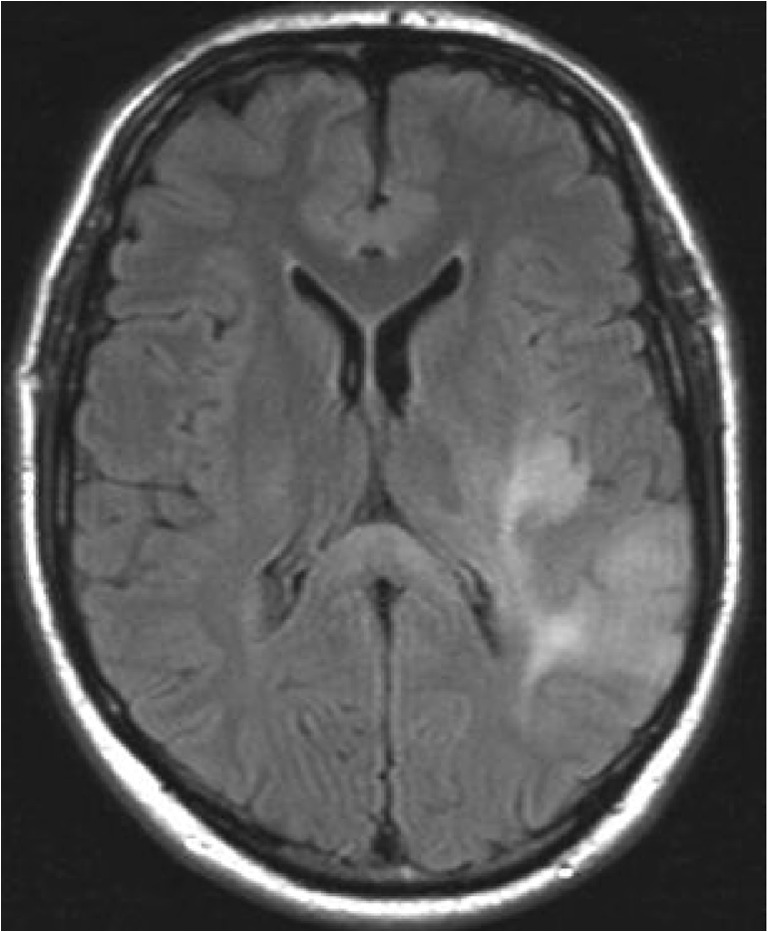
Forty-nine-year-old man with a diffuse glioma grade 2. Axial FLAIR shows a diffuse tumor with extension into the splenium, consistent with appearance of gliomatosis cerebri

In a study by Peretti-Viton et al., the CC was involved in 8 of 9 patients with gliomatosis cerebri, with splenial involvement shown in one figure [88]. Desclée et al. describe 1 of 12 patients to have extension into the splenium [89]. Kontzialis et al. show a child with diffuse glioma in the splenium [32]. In Fig. 9, an MRI scan of a male patient with a biopsy proven GBM is shown. The patient presented with light expressive aphasic disorder and periods of unresponsiveness and could no longer perform executive functions. Figure 10 shows an MRI scan of a male patient with a biopsy proven diffuse astrocytoma grade 2. This patient presented with word retrieval disturbance for several months, headache and insecure while walking. The patient passed away 9 months after the MRI.

**Fig. 11:**
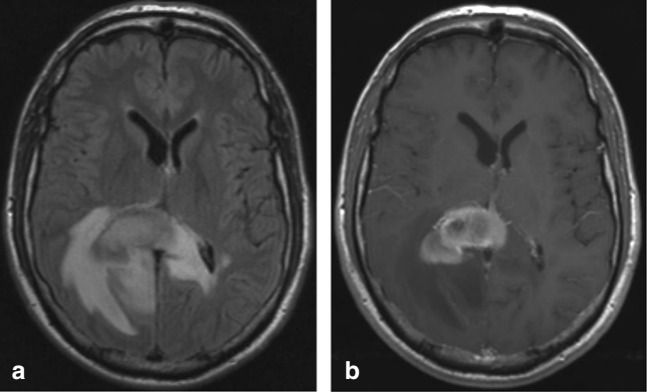
Forty-seven-year-old man with biopsy proven B cell lymphoma. Axial FLAIR (**a**) shows a space occupying lesion with a slightly hyperintense center and a hypointense rim in the splenium, which enhances vividly after contrast (axial T1 with gadolinium (**b**)

Description of symptoms in splenial involvement of GBM and gliomatosis cerebri has been limited to case reports.

Yapici-Eser et al. describe a 46-year-old women with a GBM, presenting with fatigue, and depressive symptoms, consisting of anhedonia (inability to experience pleasure) and avolition (decrease in motivation to perform self-directed purposeful activities). She also had psychomotor retardation and increased sleep. Neuropsychological testing revealed primary verbal and visual memory impairment [84].

Kiely and Twomey report a case of a 61-year-old male, with a histologically confirmed GBM involving the splenium on MRI, presenting with disorientation besides more general features of increased intracranial pressure [90].

#### *B2b. Lymphoma and hypothesis for local development in the splenium*

Primary central nervous system lymphoma (PCNSL) is a rare tumor, accounting for 2.8% of all primary brain tumors [91]. Its involvement of the CC has been reported in 14–28% [92, 93]. Splenial involvement in PCNSL has been described by Bruno et al. in 6/7 cases with TERTp-mutated PCNSL [94]. Although these and other reports only show images with splenial PCNSL, this involvement is not specifically mentioned in the text [82, 92, 93, 95]. The reason for this tendency is not understood, but may be related to microglia, T cell lymphocytes and perivascular spaces.

Approximately 90% of PCNSLs are diffuse large B cell non-Hodgkin lymphomas (DLBCLs), while primary T cell lymphomas constitute about 2% [93, 96–98]. DLBCLs are composed of immunoblasts with an angiocentric pattern. Focally prominent reactive astrocytic and microglial response is common, as well as reactive T cell lymphocytic infiltrates presence in varying degrees, with predominance of small CD4-positive T cells [93, 98].

Prinz et al. in 2014 conclude that the microglial cells derive from the multipotential hematopoietic stem cells, just like the lymphocytes involved in other forms of lymphoma [99]. Microglia cells play a role in brain development and maintenance of neuronal networks, but are also seen as the macrophages of the CNS. They differ from the “normal” macrophages elsewhere in the body, but have a similar function in eliminating microbes and dead cells [100]. High numbers of macrophages have been found in perivascular spaces. These macrophages interact with lymphocytes in the blood to promote local immune responses [101]. During inflammatory processes, activated T cells located within the perivascular spaces are able to cross the blood brain barrier and bind to microglia. These spaces therefore play an important role in lymphocyte trafficking [102] and are also referred to as the glymphatic system [101].

The greater number of perivascular spaces in the splenium may be associated with a larger number of neighboring microglia cells. In 1939, Kershman referred to del Río Hortega, who first described nests of microglia, which have also been referred to as microglial ‘fountains’ from which microglia cells are constantly projected into the brain. In addition, there are smaller foci where ameboid perivascular cells and submeningeal ameboid elements develop into mature branched microglia cells [103]. Once activated by a trigger, microglia cells change from an immunological inert state into an amoeboid round form and display behavior similar to macrophages in the blood. Amoeboid microglia cells have been demonstrated in the CC [104].

PCNSL tumor spread along perivascular spaces has been suggested by Batchelor and Loeffler [93]. They point out that enhancement in PCNSL shows a characteristic pattern, emanating from the CC. We suggest that compared with the remainder of the CC, its splenial involvement may be explained by its thicker size with a greater number of perivascular spaces surrounding perforators, related to the pericallosal pial plexus [18, 19].

Imaging characteristics of PCNSL vary widely among cases. On MRI, due to hypercellularity, lymphomas are isointense with gray matter on T2 and iso- or hypointense on T1-weighted images, with significant diffusion restriction on DWI/ADC. Contrast enhancement is intense and homogenous in about 85–95% of cases, but can be inhomogeneous or even missing. Figure 11 shows a case of a male patient presenting with leftsided homonymous hemianopia, leftsided sensory disturbances and a 3rd nerve palsy on the left, with a large cell B cell lymphoma located in the splenium.

**Fig. 12:**
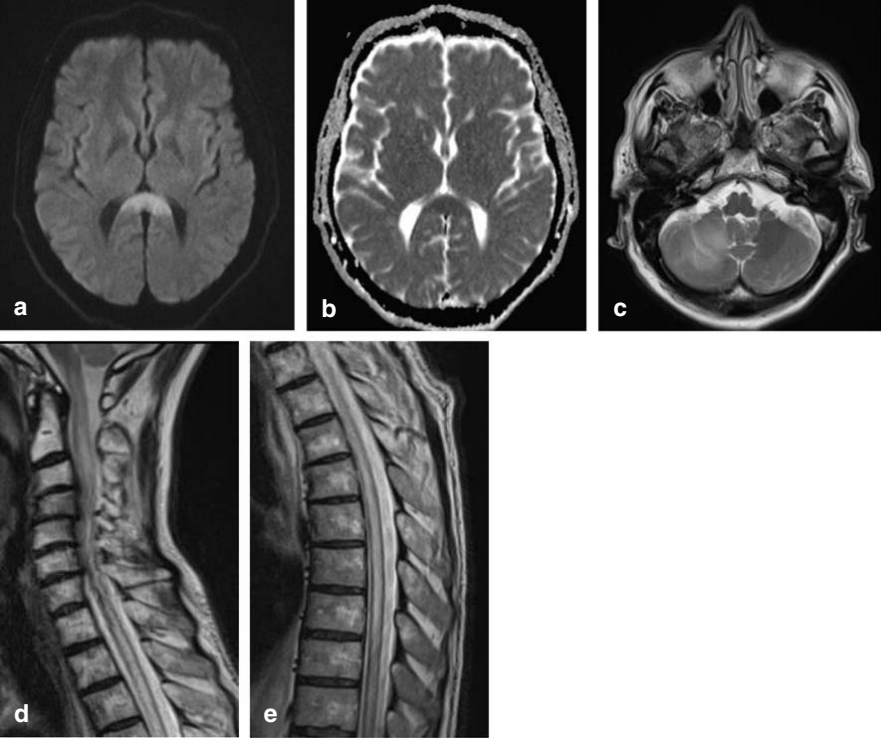
Sixty-four-year-old man with biopsy proven demyelination with some T cell infiltrate and slight secondary axonal injury, consistent with ADEM. Axial DWI (**a**) and ADC (**b**) show large splenial lesion with diffusion restriction, without enhancement (not shown). Axial T2 (**c**) shows lesion in the right cerebellar hemisphere, with central punctiform enhancement (not shown). Swelling of the entire spinal cord shown on sagittal T2 (**f**, **g**)

Clinical signs are generally rapidly progressive and non-specific. In about 50–70% personality changes and cognitive impairment are found [105], which could be related to the splenium.

#### B3. Inflammatory diseases

Many inflammatory diseases, such as MS, and neuromyelitis optica spectrum disorder and vasculitis may lead to lesions in the CC; however, they generally are not limited to the splenium and therefore are not included in this section.

#### *B3a. Tumefactive acute encephalomyelitis*

The splenium may be involved in inflammatory brain disease such as acute tumefactive demyelinating encephalomyelitis (ADEM) through local extension from a location in the adjacent parietal lobe white matter. The inflammation may be triggered by a viral infection or vaccination. On MRI, gray and white matter may be involved with focal areas of high T2 signal, with variable enhancement [106]. The splenium may be affected together with lesions elsewhere in the brain and spine. Figure 12 shows a case of an older male patient, presenting with a cerebellar syndrome, sensory disturbance and weakness in the right leg, dysarthria, eyemovement disorder and alternating confusion with delireum, followed by depression. Biopsy of the splenial lesion showed demyelination with T cell infiltrate and slight secondary axonal injury, consistent with ADEM.

**Fig. 13:**
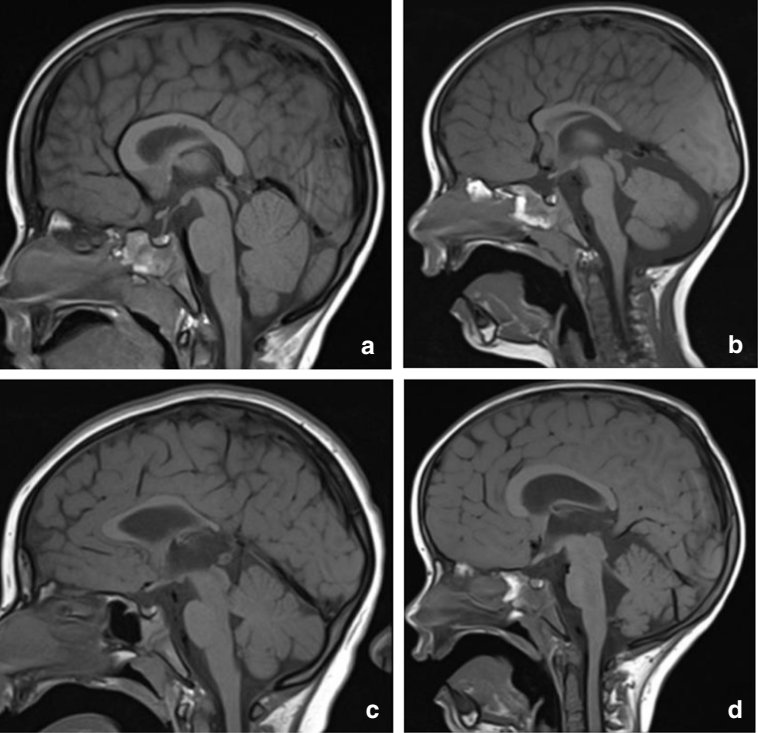
Normal control for comparison, 5-year-old boy with headaches. Normally developed CC with comparable thickness of splenium and genu (**a**). A 3.5-year old boy with a TUBB2A mutation with developmental and motor delay with splenial dysgenesis. Also presenting with developmental disturbance of the pons and cerebellum (**b**). Five-year-old boy with developmental delay, dysmorphic features, epilepsy, and spastic paraplegia, associated with an AP4S1 gene mutation, with the most severely affected volume restriction of the splenium (**c**). Five-year-old boy with progressively enlarging skull circumference associated with an mTOR gene mutation. The splenium is dysgenetic, with smaller caliber compared with the genu (**d**)

#### *B3b. Susac's syndrome *

Susac syndrome is an autoimmune endotheliopathy, known to affect the CC in females, between the ages 20–40, presenting with the classic triad of hearing loss, retinal artery occlusion and encephalopathy based on microangiopathy [107]. Although MRI shows typical “snowball lesions” representing microinfarcts throughout the entire CC [107], in multiple case reports, these lesions are shown to be present in the splenium, suggesting a preference for this area [108, 109].

Symptoms include headache, and during the encephalopathy phase early manifestations include acute altered mental status, personality changes, including depression, and are followed by memory loss and dementia [108].

### C. Congenital and acquired pathology involving the splenium in a general disorder

#### C1. Developmental disorders

#### *C1a. Agenesis and dysgenesis*

Agenesis of the corpus callosum (ACC) is one of the most common brain developmental disorders. Incidence of ACC varies among 0.5–70 in every 10,000 births [110]. In children with mental retardation, its prevalence is estimated to be up to 230 ACCs in 10,000 births. The exact prevalence of isolated splenial agenesis, however, is unknown [111]. A multitude of genetic mutations is known to affect the development of the splenium, leading to dysgenesis or agenesis. Hanna et al. provide a schematic overview of different appearances of CC dysgenesis and agenesis [112]. MRI scans of three patients with different genetic mutations associated with dysgenesis are presented in Fig. 13.

**Fig. 14:**
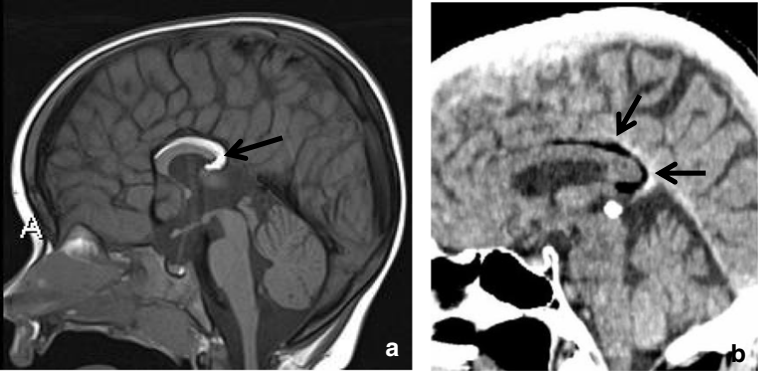
Two-year-old child. Sagittal T1 shows dysgenesis of the corpus callosum mostly affecting the splenium, with a curvilinear lipoma extending from below the splenium continuing anteriorly, covering the superior surface of the corpus up to the genu (**a**). Seventy-two-year-old man. Sagittal CT reconstruction shows a sharply demarcated hypodense structure curving posteriorly around a normal appearing splenium, consistent with a lipoma (black arrows). The bright spot inferior to the lipoma is a calcified pineal gland (**b**)

Often, splenial dysgenesis or agenesis is found in the presence of other structural anomalies in the CNS, such as microcephaly, Dandy-Walker syndrome or polymicrogyria, heterotopia and cortical dysplasia. In holoprosencephaly, an atypical CC dysgenesis has been described, with formation of the posterior part of the CC, including the splenium, and absence of the anterior portion [113].

Contradictory findings have been published on agenesis of the entire CC and development. A meta-analysis of nine studies suggests that 70% of children, prenatally diagnosed with isolated CC agenesis, develops normally [114]. However, there are also several studies which report the opposite [110, 115]. Whether clinical features are related to the absence of the splenium, or to the associated bilateral hippocampal malrotation is unknown.

A review study by Chiarello has compared behavioral strategies and performance of spatial and linguistic functions in patients with ACC and split brain patients. She describes that callosal agenesis is often accompanied by spatiomotor function impairment. On the contrary, commisurectomy cases are unable to perform tasks requiring integration or intrahemispheric transfer of information, such as verbal response to leftsided visual input [116], presumably due to splenial involvement.

#### *C1b. Lipoma*

Intracranial lipomas are rare congenital malformations, which are believed to result from abnormal persistence and maldifferentiation of the primitive meninges during development of the subarachnoid cisterns [117]. Others report that they account for 0.46–1% of intracranial tumors [118]. Intracranial lipomas mainly occur at or near the midline, in the region of the pericallosal cistern and the CC, with curvilinear lipomas sweeping around a dysplastic splenial segment [1]. Agenesis or dysgenesis of the CC is the most frequently associated brain anomaly [119].

On T1-weighted MR images, they are very hyperintense, as shown in Fig. 14a, which shows the scan of a child with a SOX 2 gene mutation and developmental delay, particularly affecting speech and motor development. On CT, lipomas are extremely hypodense with a very low Hounsfield unit value. This is shown in Fig. 14b demonstrating a CT scan made of an older patient scanned for neurological deficit related to a cerebrovascular attack, with a coincidental finding of a lipoma curving along a normal splenium.

**Fig. 15:**
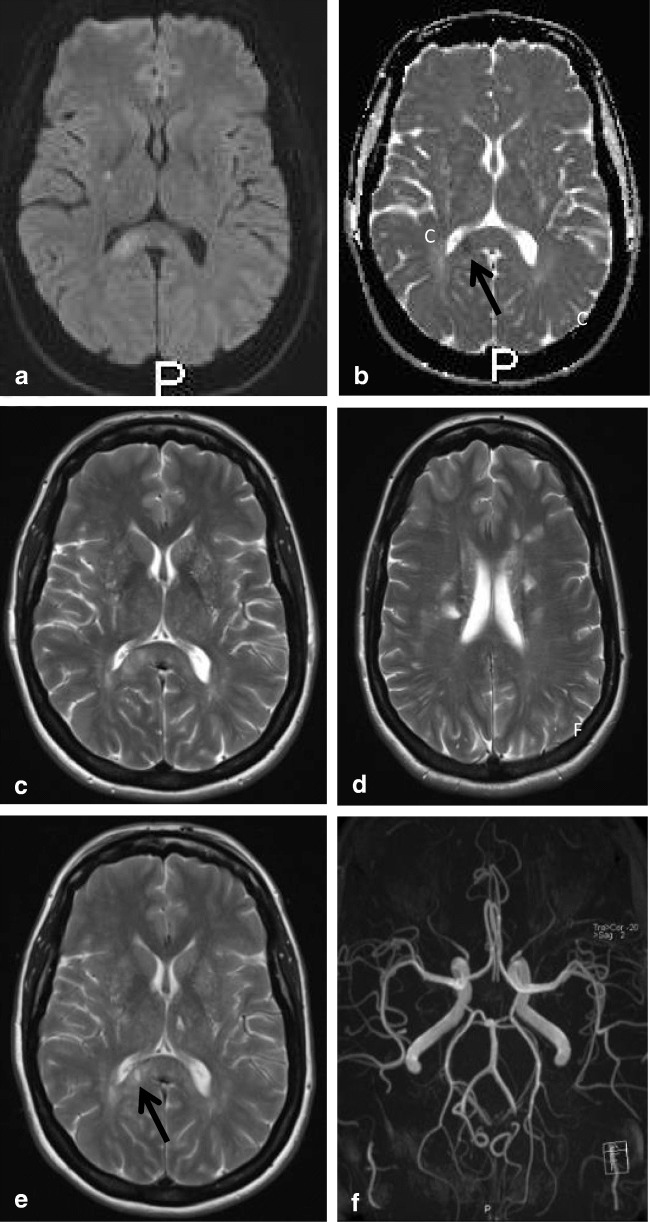
Forty-eight-year-old woman. Axial DWI (**a**) and ADC (**b**) show a small area of diffusion restriction in the right splenium (black arrow) with a larger area of edema on the T2 (**c**). In the corona radiata and frontal white matter, multiple lacunar infarcts are seen (**d**). Follow-up axial T2 (**e**) at the level of figure **c** shows residual focal loss of tissue in right splenium and a similar lesion in left thalamus (black arrow). 3D TOF MRA shows normal cerebral arteries (**f**)

Most intracranial lipomas are asymptomatic and considered an incidental finding on imaging.

#### *C1c. Infarction and hypoxic ischemic encephalopathy*

Infarctions restricted to the CC are rare, because of its rich blood supply from the different arteries bilaterally. Furthermore, the perpendicular orientation of the callosal branches may prevent embolization [120]. If infarction is restricted to the CC, it is mostly found in the splenium [19, 120]. Splenial infarction may be considered a watershed area, because of the anastomosis between the endbranches of the anterior and posterior communicating arteries. Isolated occlusion of these arteries does not necessarily lead to interruption of blood supply to the splenium with subsequent infarction [120]. In Fig. 15a, b, a DWI/ADC map shows an area of cytotoxic edema, compatible with acute ischemia in the right side of the splenium, in a female patient presenting with acute dysarthria, without cognitive disturbance. The scan shows older lacunar infarcts in the corona radiata and left frontal white matter during the acute phase (Fig. 15c, d). On follow-up, a small residual lacune is seen in the area of cytoxic edema seen during the acute phase, in addition to a lacune in the left thalamus (Fig. 15e). No abnormalities were seen on the MRA (Fig. 15f).

**Fig. 17:**
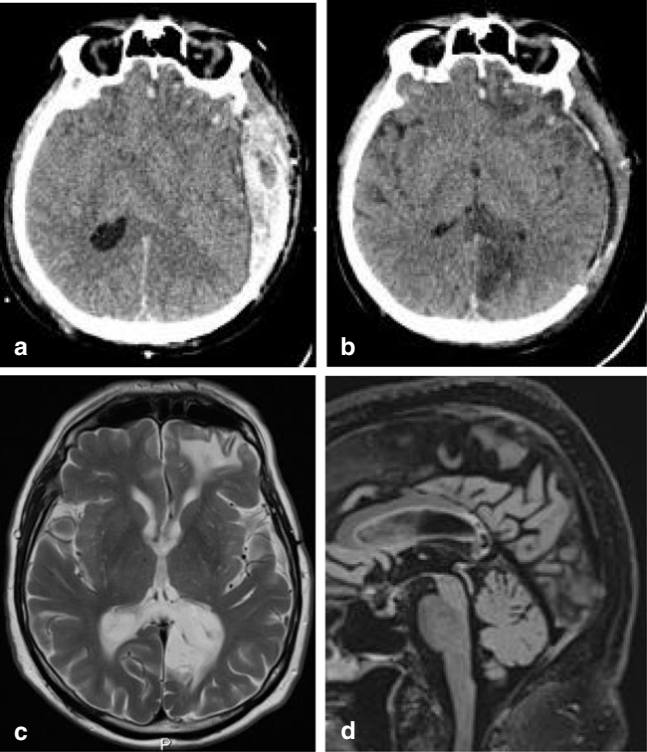
Sixty-six-year-old man. Axial CT with leftsided subdural hematoma and diffuse brain swelling without splenial hypodensity on day of trauma (**a**); 3 days later, infarction is seen in the left side of the splenium and in the left occipital lobe (**b**). At follow-up, 7 years later, the patient has no residual cognitive disturbance. Axial T2 (**c**) and sagittal 3D FLAIR (**d**) show extreme tissue loss in the same regions

In neonates with hypoxic ischemic encephalopathy (HIE), parietal-occipital infarction may lead to a secondary involvement of the splenium with diffusion restriction on DWI, compatible with cytotoxic edema. FA decrease may be found, suggesting loss of myelinated axonal integrity [121, 122].

Alderliesten et al. described an adverse neurological outcome in term neonates with diffusion restriction in the posterior CC [123]. However, most children also have abnormalities elsewhere and it is difficult to know which clinical signs could be attributed to the splenial involvement alone.

#### *C1d. Traumatic lesions*

**Fig. 16:**
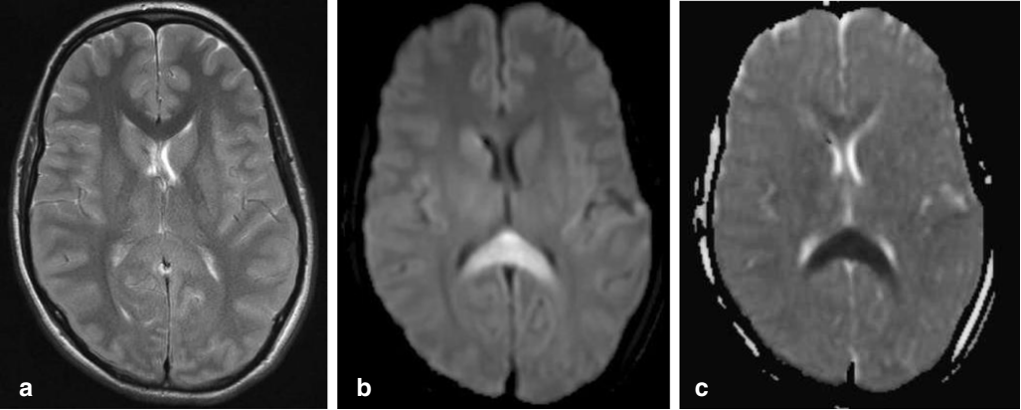
Fifteen-year-old girl post high energy  trauma. CT on day of trauma (not shown) showed no abnormalities. Axial T2 MRI (**a**) at day 6, post trauma, shows involvement of the entire splenium, with restricted diffusion on DWI (**b**) and ADC map (**c**). At 6-week follow-up, the patient had severe residual cognitive disturbance

During trauma, the CC is restricted in left to right movement because of its narrow relationship with the posterior falx. The splenium has an additional movement restriction in caudo-cranial direction because of the tentorium below. This leads to an increased susceptibility to traumatic injury [124, 125]. After high energy trauma, it is considered part of diffuse axonal injury [126, 127]. In the acute phase, diffusion restriction may been seen on DWI, as shown in Fig. 16, made of a child after high energy trauma with GCS score 1-2-1. Microhemorrhages may be seen on susceptibility weighted imaging (not shown). At follow up these lesions often show focal tissue loss with CSF signal due to disruption of crossing fibers. Figure 17a  illustrates a large acute hyperdense subdural hematoma with brain swelling and clear midline shift on a CT scan, made following trauma in an older male patient, presenting initially with paralysis of the right arm and aphasia, with subsequent hemianopia and disorientation post craniectomy. The midline shift could potentially compress the splenial artery against the tentorium, leading to focal infarction as is shown on the postcraniectomy CT in figure 17b and follow up MRI scan in figure 17c-d (see section on Vascularization and Infarction) [124].

**Fig. 18:**
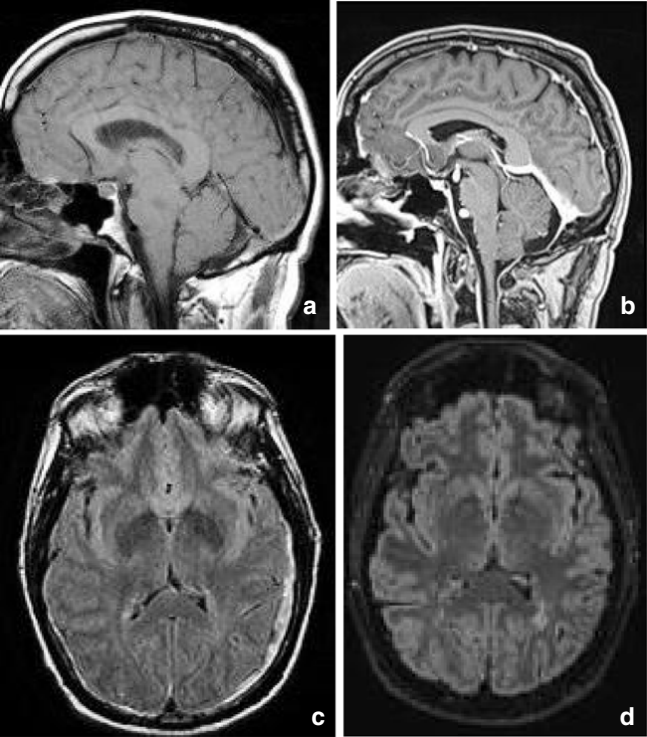
Fifty-three-year-old man with intracranial hypotension syndrome. At first admission, sagittal T1 (**a**) shows an enlarged and downward herniating splenium, with normal signal intensity on the axial FLAIR (**c**). Also sagging midbrain and bilateral subdural hematoma. Seven years later scanned for left frontal headache, disappearing with reclining. Sagittal T1 after gadolinium (**b**) shows normalized position brainstem, with decreased enlargement of the splenium. On axial FLAIR (**d**), no new SDH is seen

Clinically, acute severing of the crossing fibers following head trauma is highly associated with poor functional outcome [126, 127] and often leads to hampering of regaining consciousness (see paragraph Function).

#### *C1e. Splenium in intracranial hypotension syndrome*

Intracranial hypotension is thought to result from CSF hypovolemia due to a spinal CSF leak. To compensate, several mechanisms come into play and can be seen on MRI: downward displacement of the brain with sagging brainstem, pituitary volume increase, dilatation of veins and dural sinuses, thickening and enhancement of the dura and sometimes subdural hematomas [128, 129]. Another sign which has been described is a particular shape and position of the splenium, which appears stumpy and displaced downwards [129]. This is well demonstrated in Fig. 17 showing an MRI scan made of a male patient, presenting with headache in upright position, which decreased with reclining. Clinically, position-dependent headache, worsening with upright position is referred to as orthostatic headache. Atypical presentations reported in literature include short-term memory problems and a not-otherwise specified subcortical type of cognitive impairment, obtundation, stupor, drowsiness and even coma [130]. Although the compressions of the midbrain tegmentum and brainstem impression have been implicated in some of the clinical signs [128], we suggest that disturbed consciousness and memory could be attributed to splenial swelling and/or herniation (Figure 18).

## Conclusion

We have presented an overview of development and function of the splenium and of pathology primarily affecting the splenium, extending into or from the splenium and involving the splenium in a general disorder. We have suggested hypotheses for the predilection of certain diseases and disorders for the splenium and for the associated clinical presentation. In acquired pathological processes, the splenium appears to play a role in consciousness.

## Abbreviation

AC: anterior commissure
ACC: agenesis of the corpus callosum
ADEM: acute disseminated encephalomyelitis
ADH: anti-diuretic hormone
AED: anti-epileptic drugs
AVP: arginine-vasopressin
CC: corpus callosum
CLOCC: cytotoxic lesions of the corpus callosum
DLBCL: diffuse large B cell lymphoma
GBM: glioblastoma multiforme
HC: hippocampal commissure
HIE: hypoxic ischemic encephalopathy
MERS: mild encephalitis/encephalopathy with a reversible splenial lesion
PCNSL: primary central nervous system lymphoma
RESLES: reversible splenial lesions syndrome
